# Molecular Mechanisms of *Panax japonicus* var. *major* Against Gastric Cancer: Metabolite Analysis, Signaling Pathways, and Protein Targets

**DOI:** 10.3390/ph18060823

**Published:** 2025-05-30

**Authors:** Chao Huang, Ge Li, Xiuxiang Yan, Terd Disayathanoowat, Angkhana Inta, Lu Gao, Lixin Yang

**Affiliations:** 1 School of Ethnic Medicine, Yunnan Minzu University, Kunming 650504, China; skt22110@outlook.com (C.H.); lllyy18@126.com (G.L.); 2 Key Laboratory of Economic Plants and Biotechnology, Kunming Institute of Botany, Chinese Academy of Sciences, Kunming 650201, China; yanxiuxiang@mail.kib.ac.cn; 3 Department of Biology, Faculty of Science, Chiang Mai University, 239 Huay Kaew Road, Chiang Mai 50200, Thailand; terd.dis@gmail.com (T.D.); aungkanainta@hotmail.com (A.I.); 4 Research Center of Deep Technology in Beekeeping and Bee Products for Sustainable Development Goals (SMART BEE SDGs), Chiang Mai University, 239 Huay Kaew Road, Chiang Mai 50200, Thailand; 5 Southeast Asia Biodiversity Research Institute, Chinese Academy of Sciences, Yezin, Nay Pyi Taw 05282, Myanmar

**Keywords:** *Panax japonicus* var. *major*, multiple pathways and targets, metabolite profiling, anti-gastric cancer activities, molecular mechanisms

## Abstract

**Background/Objectives:** Ginseng (*Panax japonicus* var. *major*) is a traditional medicinal plant with anticancer properties. We aimed to assess the biological activity, potential targets, and molecular mechanisms of *P. japonicus* var. *major* in resisting gastric cancer. **Methods:** We developed a model that combines network pharmacology, molecular docking, untargeted metabolomics, and molecular dynamics simulations to predict which compounds from *P. japonicus* var. *major* might be active in the treatment of gastric cancer. We conducted in vitro experiments and immunoblot validation to test these predictions. **Results:** We identified 44 main active compounds from *P. japonicus* var. *major* and 29 core targets. These compounds showed anti-gastric cancer activity against the HGC-27 cell line by acting on TNF and T-cell receptor signaling pathways to diminish inflammatory factor production and promote apoptosis of gastric cancer cells. Clinical survival analysis identified four core proteins (CASP3, TNF, AKT1, and EGFR) whose abundance was associated with survival status in gastric cancer patients. Molecular docking, along with molecular dynamics simulations, revealed that these core proteins could be stably bound by the identified active compounds. The anti-gastric cancer effects of *P. japonicus* var. *major* compounds involved a lower Bcl-2/Bax ratio and upregulation of CASP3 and CASP9 levels, highlighting significant differences in anti-gastric cancer activity between extracts prepared from fresh versus dried *P. japonicus* var. *major*. **Conclusions:** Our results provide background for the indigenous medicinal use of *P. japonicus* var. *major* to treat gastric cancer and lay the foundation for further pharmacological experiments and clinical tests.

## 1. Introduction

Gastric cancer (GC) is a major type of malignant tumor and a leading cause of death worldwide, ranking fifth in terms of incidence and mortality rates; notably, the incidence of GC among young people is on the rise [1]. GC patients who cannot be treated through surgery have no other treatment options, and the survival rate of patients with advanced GC is only 20% [2]. Therefore, it is urgent to find drugs that can treat GC or be used as adjuvant therapy.

A non-targeted metabolomics approach was previously used to investigate the anticancer and immunomodulatory effects of fermented leaves from Korean perilla (*Perilla frutescens*) [3]. A combination of network pharmacology and molecular docking experiments clarified the active ingredients from the fungus *Armillaria ostoyae* that contribute to GC treatment and the related pathways they may target [4]. Molecular docking and clinical medical research can be combined to help predict the mechanisms of drug-activated diseases and improve the therapeutic efficacy of drug-activated lesions [5].

The biological pathways and regulatory genes targeted by bioactive compounds from medicinal plants have recently been studied for the treatment of GC. Several medicinal plants possess anti-GC properties, for example, *Paris polyphylla* [6]; *Radix Astragali*, the root of *Astragalus membranaceus* var. *mongholicus* [7]; *Pseudostellaria heterophylla* (Miq.) *Pax* [8]; and *Curcuma longa* L. [9]. The natural antitumor drugs with clinical applications are mainly alkaloids [10], terpenoids [11], polyphenols [12], and polysaccharides [13]. Most of these compounds display high efficacy in treating GC. For instance, treatment with Taxol (paclitaxel), which interferes with the microtubules of GC cells, blocks mitotic progression and interrupts cell growth, thus inducing the shrinkage of GC cells and eventually apoptosis [14]. Alkaloids can activate crosstalk between apoptosis and autophagy by inhibiting the phosphoinositide 3-kinase (PI3K)/Akt/mammalian target of rapamycin (mTOR) pathway and thus repress the viability of GC cells [15]. Moreover, an alkaloid derived from the marine fungus *Arthrinium arundinis* inhibits the growth and metastasis of GC cells by targeting the mTORC1 signaling pathway [16]. Much progress has been made in the study of natural antitumor compounds, but Taxol is one of the few known to have significant antitumor efficacy. More research on natural antitumor compounds is needed to explore the integration of biological pathways and genes affected by these chemicals.

*Panax japonicus* var. *major*, from the Araliaceae family, is a species of ginseng found mainly in the Yunnan province of China, where it has long been used as a natural medicine [17]. The roots of this traditional Chinese medicinal plant contain 122 compounds that have been isolated and identified, mainly consisting of saponins and flavonoids [18]. Total saponins from *P. japonicus* var. *major* possess anticancer, immunomodulatory, anti-inflammatory, analgesic, cardioprotective, hepatoprotective, and antioxidant activities [19,20,21,22,23]. Indeed, extracts from *P. japonicus* var. *major* have been used to treat inflammation, hepatocellular carcinoma, ischemic brain damage, and other diseases [24].

**Figure 1:**
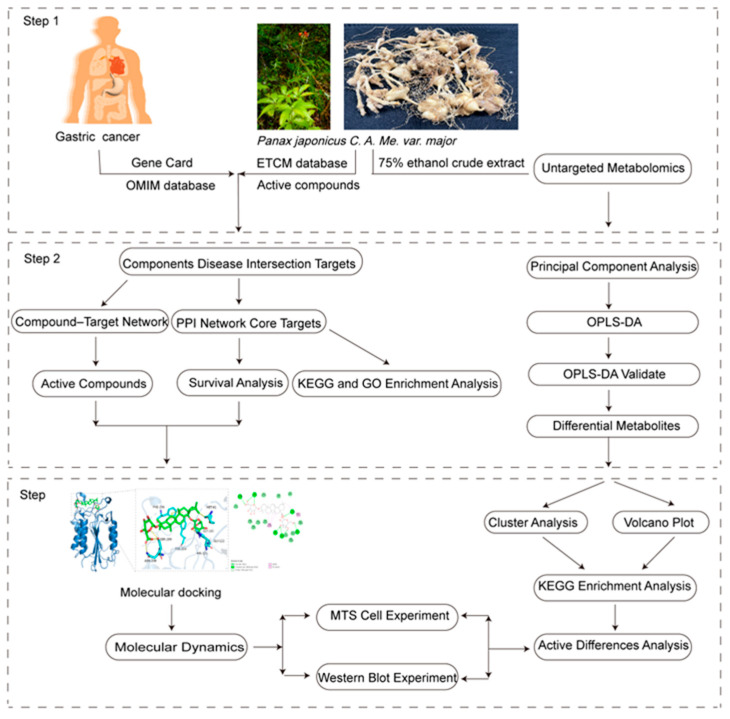
Analysis workflow of network analysis and untargeted metabolomics.

We reasoned that *P. japonicus* var. *major* extracts might be useful for the treatment of GC. We investigated the effects of *P. japonicus* var. *major* on GC and associated mechanisms, starting with the identification of the main active compounds in plant extracts and their cellular targets (Figure 1). We further assessed the therapeutic efficacy and mechanism of action of *P. japonicus* var. *major* extracts for the treatment of GC. This study provides a theoretical basis for the deployment of natural medicinal herbs for GC treatment.

## 2. Results

### 2.1. Screening for Active Ingredients of P. japonicus var. major and Gastric Cancer Targets

We first obtained a list of 44 known active compounds present in *P. japonicus* var. *major* extracts from the ETCM database (Figure S1). These active compounds were mainly saponins with anticancer and anti-inflammatory activities, including chikusetsu saponin IVa; the ginsenosides Ro, Rc, and Re; taibaienoside I; panaxjapyne A; panaxjapyne B; and panaxjapyne C, [25,26]. We then predicted the cellular targets of these 44 compounds using the Swiss Target Prediction tool, which identified 355 potential targets for these active compounds. Separately, we assembled a list of 2103 GC targets by screening the Gene Cards and OMIM databases with the keyword “gastric cancer”. Comparison of the 355 predicted targets for active compounds from *P. japonicus* var. *major* and the 2103 GC targets defined a set of 125 common targets.

### 2.2. Main Components and Targets Associated with Gastric Cancer

**Figure 2:**
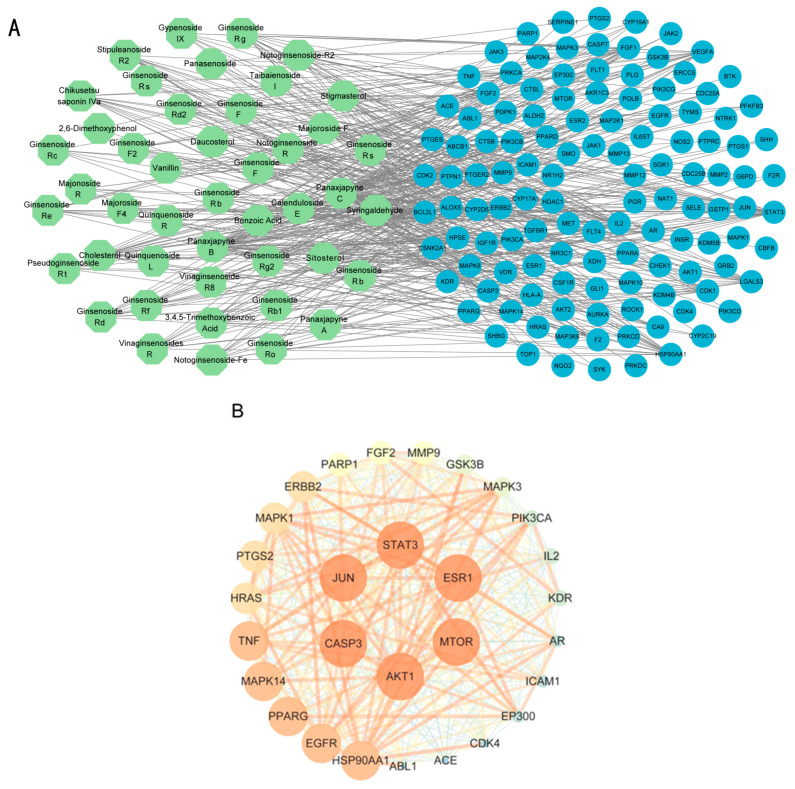
Identification of key active ingredients and core targets through network pharmacology. (**A**) Compound–target network. Green nodes represent active compounds, and blue nodes represent targets. (**B**) Protein–protein interaction network.

To examine how these 125 predicted common targets interact, we constructed a component–target map and the underlying protein–protein interaction (PPI) network (Figure 2A). We screened each main active ingredient and their corresponding targets and ranked them according to their degree, betweenness, and closeness values in the network (degree represents the number of direct interactions of a molecule; betweenness indicates the importance of a molecule as a transit station for information transmission; closeness measures how close a node is to all other nodes in the network in terms of average distance). We chose the top ten active compounds based on their degree values for analysis (Table 1): panaxjapyne C, panaxjapyne B, cholesterol, β-sitosterol, stigmasterol, notoginsenoside R2, 3,4,5-trimethoxybenzoicacid acid, chikusetsu saponin IVa, stipuleanoside R2, and calenduloside E. We surmised that focusing on high-degree nodes can potentially produce broader therapeutic effects by influencing multiple pathways simultaneously.

Separately, we drew a PPI network in Cytoscape 3.10.2 for all 125 common targets and their interactors based on data obtained from the STRING database (Figure 2B). The visualization of the PPI network used the following settings: closeness > 0.046, betweenness > 99.70, degree > 33.07. The top 29 targets for treatment of GC, based on decreasing degree values, are listed in Table 2; these targets are potentially associated with GC.

### 2.3. Potential Functions and Pathways Associated with Targets for GC Treatment

**Figure 3:**
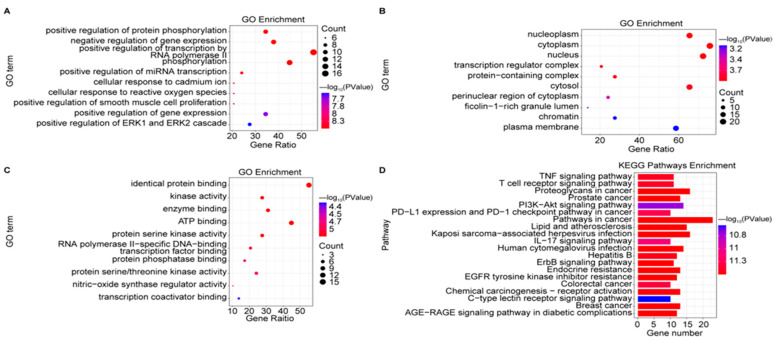
KEGG and GO enrichment analyses of all 29 core targets. (**A**–**C**) GO term enrichment for (**A**) biological processes, (**B**) cellular components, and (**C**) molecular function. (**D**) KEGG pathway enrichment analysis.

We explored the potential mechanisms and pathways enriched among the common targets by conducting gene ontology (GO) term and Kyoto Encyclopedia of Genes and Genomes (KEGG) pathway enrichment analysis of the 29 core targets. We identified 313 terms related to biological process (BP), 34 terms related to cellular component (CC), 54 terms related to molecular function (MF), and 141 KEGG pathways as being enriched; the top 10 terms or pathways are shown as bubble diagrams in Figure 3. The predicted effects of compounds derived from *P. japonicus* var. *major* were mainly related to ‘negative regulation of gene expression’ and ‘positive regulation of protein phosphorylation’ (Figure 3A) among the top 10 BP terms. The common targets were mostly predicted to localize to the cytoplasm and the nucleus (Figure 3B). The MF terms enriched in common targets (Figure 3C) were ‘identical protein binding’, ‘enzyme binding’, and ‘ATP binding’.

**Figure 4:**
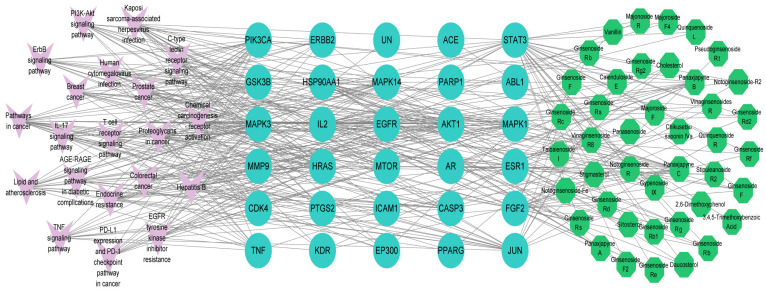
Component–pathway–target network of *P. japonicus* var. *major* active compounds and their potential targets. The green nodes represent compounds, the purple nodes indicate pathways, and the blue nodes represent core targets.

The top 20 enriched KEGG pathways are shown in Figure 3D. Three KEGG pathways highly enriched in common targets were associated with apoptosis, including the tumor necrosis factor (TNF) signaling pathway, the T-cell receptor signaling pathway, and the interleukin 17 (IL-17) signaling pathway, which are also closely associated with triggering inflammation. We also constructed a component–pathway–target diagram (Figure 4) by combining the top 20 KEGG pathways, 29 core GC targets, and 44 main active compounds. We determined that these 44 chemical compounds are associated with the core targets caspase 3 (CASP3), TNF, and the kinase AKT1, which function primarily in the TNF and p53 signaling pathways in the treatment of GC.

### 2.4. Effect of the Expression of CASP3, AKT1, TNF, and EGFR on Patient Survival

**Figure 5:**
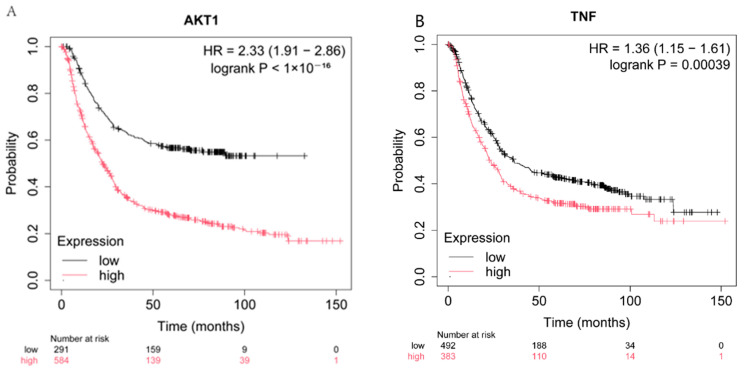

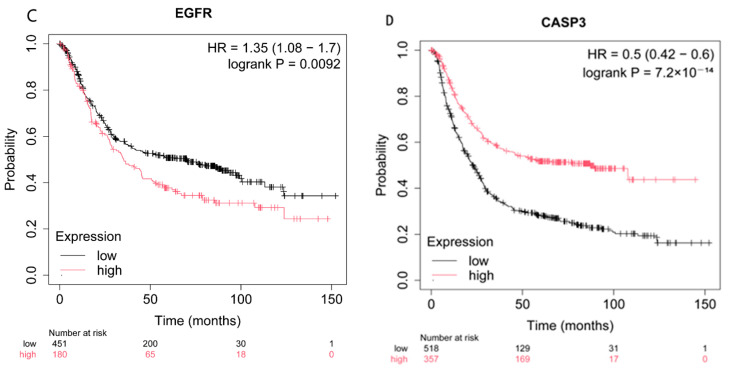
Survival curves for gastric cancer patients as a function of expression levels for four target genes. (**A**) *AKT1*. (**B**) *TNF*. (**C**) *EGFR*. (**D**) *CASP3*. The probability of survival following GC diagnosis is shown for patients with low (black curves) or high (red curves) expression for the indicated gene.

We looked for an association between clinical patient survival and the expression of each of the top four core target genes, *CASP3*, *AKT1*, *TNF*, and *EGFR*. Specifically, we generated Kaplan-Meier plots to assess patient survival at *p* < 0.05 (Figure 5). When either *AKT1*, *TNF*, or *EGFR* was expressed at a low level, patient survival was higher than when the gene was expressed at a higher level (Figure 5A–C). By contrast, higher *CASP3* expression was associated with patient survival (Figure 5D).

### 2.5. Binding Ability Between Main Active Compounds and Protein Targets

**Figure 6:**
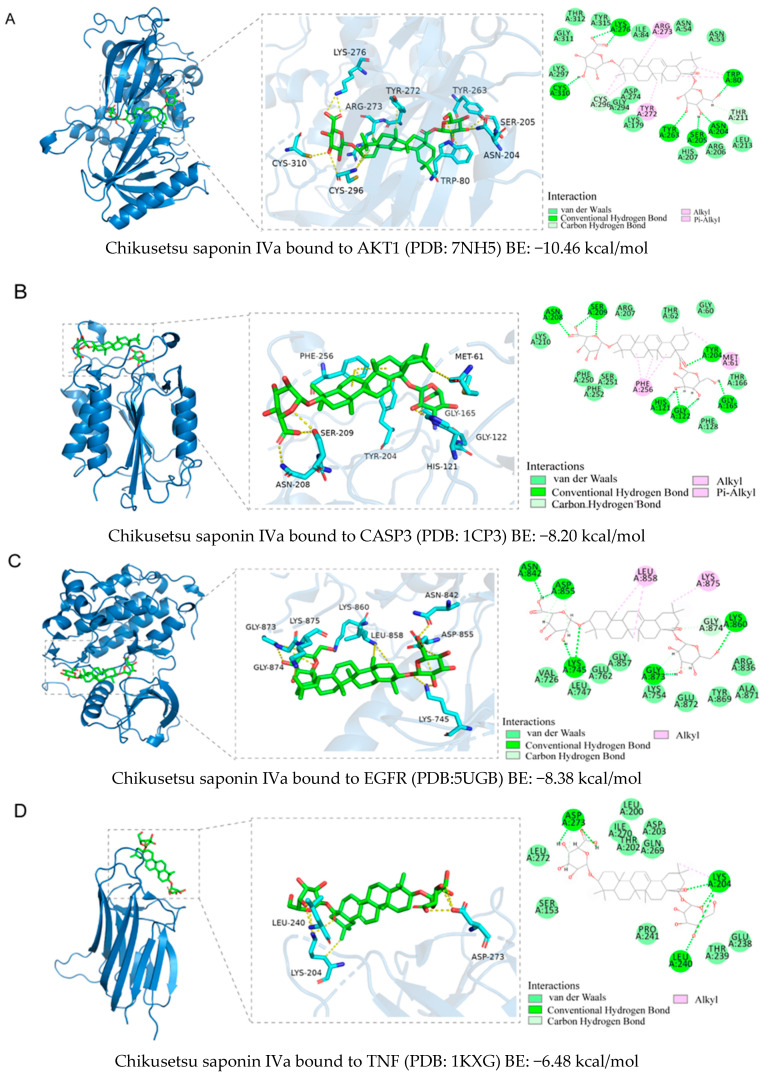

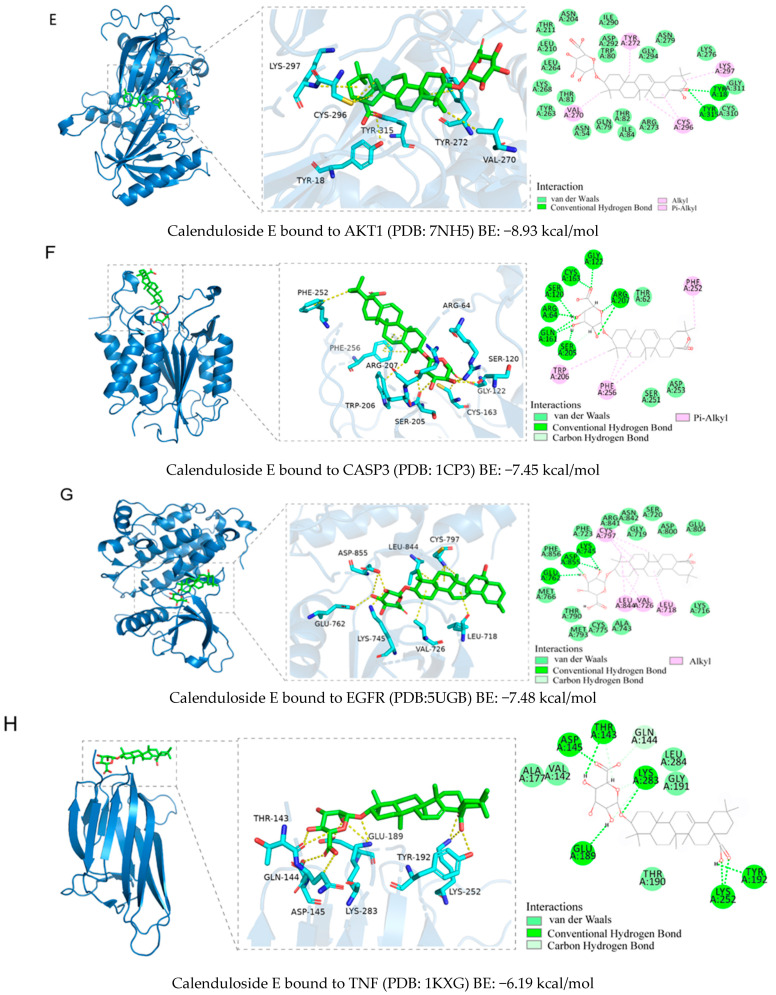
Molecular docking between the two active compounds Chikusetsu saponin IVa and calenduloside E, and the four target proteins, AKT1, CASP3, EGFR, and TNF. (**A**–**H**) Prediction of molecular docking of (**A**) chikusetsu saponin IVa onto AKT1 (PDB: 7NH5), (**B**) chikusetsu saponin IVa onto CASP3 (PDB: 1CP3), (**C**) chikusetsu saponin IVa onto EGFR (PDB: 5UGB), (**D**) chikusetsu saponin IVa onto TNF (PDB: 1KXG), (**E**) calenduloside E onto AKT1 (PDB: 7NH5), (**F**) calenduloside E onto CASP3 (PDB: 1CP3), (**G**) calenduloside E onto EGFR (PDB: 5UGB), and (**H**) calenduloside E onto TNF (PDB: 1KXG).

To examine the potential effects of *P. japonicus* var. *major* active compounds (chikusetsu saponin IVa and calenduloside E) on the four proteins identified above, we subjected these proteins (CASP3, AKT1, TNF, and EGFR) to a molecular docking analysis using MOE2019 software [27]. The analysis indicated that chikusetsu saponin IVa and calenduloside E can each access the binding pockets of all four proteins, with molecular docking energies ranging from −6.19 to −10.46 kcal/mol for these eight compounds–target pairs. Of the eight pairs, six (chikusetsu saponin IVa–AKT1, chikusetsu saponin IVa–CASP3, chikusetsu saponin IVa–EGFR, calenduloside E–AKT1, calenduloside E–CASP3, and calenduloside E–EGFR) showed strong binding activity, whereas the remaining two pairs (chikusetsu saponin IVa–TNF, calenduloside E–TNF) had weaker binding activity. The details of molecular docking between the compounds and the targets are shown in Figure 6. For example, residues Trp-80, Ser-205, Asn-204, Tyr-263, Lys-276, and Cys-310 of AKT1 formed hydrogen bonds with chikusetsu saponin IVa, whereas residues Tyr-272, Arg-273, and Cys-296 formed hydrophobic interactions. Residue Cys-296 of AKT1 formed a hydrocarbon interaction with chikusetsu saponin IVa (Figure 6A). His-121, Gly-122, Gly165, Tyr-204, Asn-208, and Ser-209 of CASP3 formed hydrogen bonds with chikusetsu saponin IVa; Phe-256 and Met-261 formed hydrophobic interactions; and the Gly-122 and Asn-208 residues formed hydrocarbon interactions (Figure 6B). Lys-745, Asn-842, Asp-855, Lys-860, and Gly-873 of EGFR formed hydrogen bonds with chikusetsu saponin IVa (Figure 6C). Lys-204, Leu-240, and Asp-273 of TNF formed hydrogen bonds with chikusetsu saponin IVa (Figure 6D). Val-270, Tyr-272, Cys-296, and Lys-297 of AKT1 formed hydrophobic interactions with calenduloside E (Figure 6E). Arg-64, Ser-120, Gly-122, Cys-163, and Ser-205 of CASP3 interacted with calenduloside E by forming hydrogen bonds (Figure 6F). Leu-718, Val-726, Cys-797, and Leu-844 of EGFR formed hydrophobic interactions with calenduloside E (Figure 6G). Finally, Thr-143, Asp-145, Tyr-192, Glu-189, Lys-252, and Lys-283 of TNF formed hydrogen bonds with calenduloside E (Figure 6H). Importantly, the docking energy values of all compounds were less than −5.0 kcal/mol, indicating strong binding to each of their targets.

### 2.6. Binding Energy and Stability of Chikusetsu Saponin IVa–CASP3

**Figure 7:**
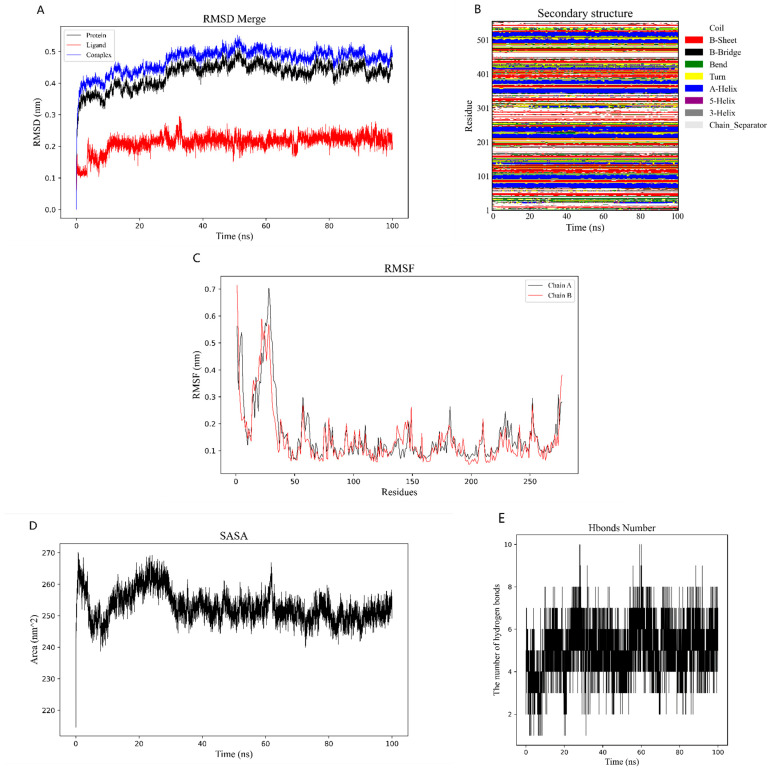
Molecular dynamics simulations. (**A**) Root-mean-square deviation (RMSD) values as a function of time. (**B**) Secondary structure. (**C**) Root-mean-square fluctuation (RMSF) values along the protein sequence. (**D**) Solvent-accessible surface area (SASA) values of complexes as a function of time. (**E**) Number of H-bonds.

We assessed the interaction strength of chikusetsu saponin IVa in complex with CASP3 through molecular dynamics (MD) simulations. We used root-mean-square deviation (RMSD) values as indicators of structural changes in CASP3 [28] and chikusetsu saponin IVa (Figure 7A). This analysis indicated that the first 20 ns of binding between CASP3 and the small molecule involve a large change in RMSD value, with an MD simulation of >100 ns. For RMSD values < 0.5 Å, the CASP3–chikusetsu saponin IVa complex, the chikusetsu saponin IVa ligand, and CASP3 showed average values of 0.43, 0.21, and 0.45 Å, respectively. During the first 20 ns of simulation, the RMSD values between the protein and the small molecule were relatively large, but gradually stabilized afterward. Overall, the RMSD values remained within a small range, indicating that the binding between the protein and the small molecule was relatively stable. These results suggest that CASP3 is structurally stable when bound to chikusetsu saponin IVa.

To verify the stability of the CASP3 secondary structure, we calculated the change in secondary structure by MD simulations. The secondary structure of CASP3 remained essentially stable in MD simulations in the first step (Figure 7B). We then calculated RMSF values between CASP3 and chikusetsu saponin IVa to identify localized fluctuations in CASP3 structure. The root-mean-square fluctuation (RMSF) values of the protein were smaller in the bound portion and larger in the unbound portion, indicating that the binding of the small molecule had some effect on the stability of the protein (Figure 7C).

In MD simulations, we calculated the solvent-accessible surface area (SASA) value for carbon (C) in CASP3 to analyze the change in surface area before and after binding of chikusetsu saponin IVa to CASP3. The SASA value was relatively larger before CASP3 bound to chikusetsu saponin IVa and then decreased and stabilized after binding (Figure 7D), indicative of a decrease in the surface area of CASP3 concomitant with an increase in the number of H-bonds between CASP3 and chikusetsu saponin IVa (Figure 7E).

We calculated the binding free energy of CASP3 in complex with chikusetsu saponin IVa to assess the stability of the complex. Specifically, we calculated the Molecular Mechanics Poisson–Boltzmann Surface Area (MMPBSA) [29]; the results are shown in Table 3.

**Figure 8:**
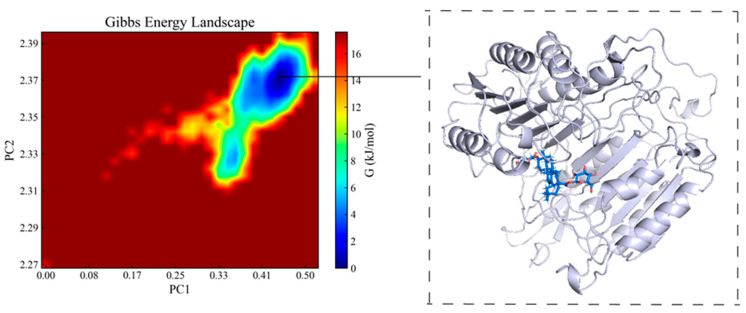
Free energy conformational map of CASP3. At left, blue indicates lower energy, red indicates higher energy; note the minimum energy point for the molecular conformation of chikusetsu saponin IVa, whose structure is shown at right.

Free energy landscape diagrams can reflect interactions and energy distribution between systems [30] and can also help clarify the interactions between small molecules and proteins, along with possible changes in conformation. Thermodynamically more stable conformations usually correspond to regions of lower free energy, whereas less stable conformations correspond to regions of higher free energy. The complex between CASP3 and chikusetsu saponin IVa had an energy minimum mainly concentrated around 99 ns during the 100 s duration of the MD simulation (Figure 8). The complex was relatively compact in this low-energy region, and the structure appeared to be able to remain stable in this state for some time.

### 2.7. Analysis of Major Components and Differential Metabolites in Panax japonicus

**Figure 9:**
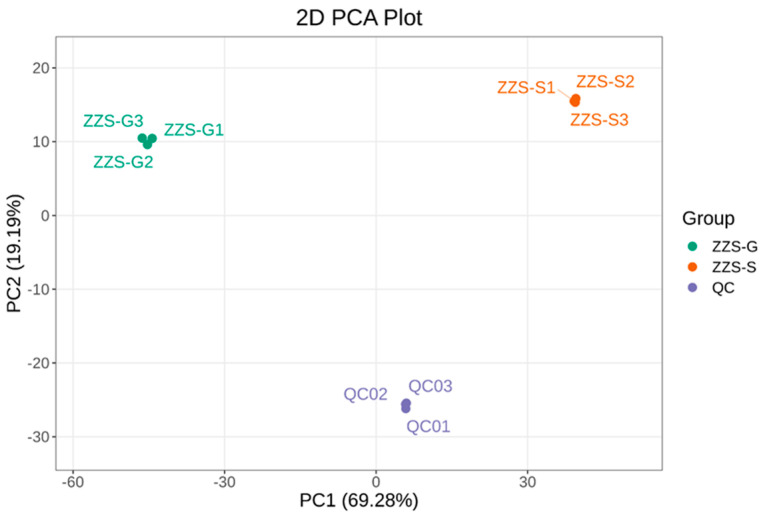
Principal component analysis (PCA) plot of *Panax japonicus* var. *major* metabolites. Each point represents an individual sample, and samples from the same group are colored the same. ‘Group’ represents the corresponding experimental grouping. PC1 and PC2 represent the first and second principal components, respectively, and their corresponding contribution rates represent the percentage of variance explained in the data.

**Figure 10:**
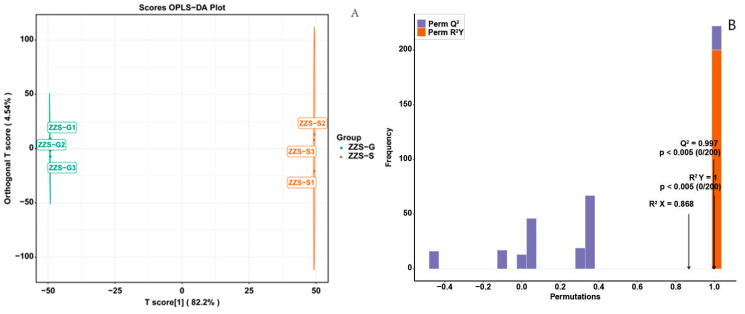
Partial least squares discriminant analysis of metabolites from *Panax japonicus* var. *major* extracts. (**A**) OPLS-DA plot. Each point represents one sample, and samples of the same group are represented by the same colors; ‘Group’ refers to the corresponding sample category. (**B**) Permutation test plot, with the *x*-axis representing the *R*^2^*Y* and *Q*^2^ values of the model, and the *y*-axis representing the frequency of classification accuracy across 200 random permutations. Orange and purple bars indicate *R*^2^*Y* and *Q*^2^ values from the permuted models, respectively, with black arrows indicating the *R*^2^*X*, *R*^2^*Y*, and *Q*^2^ values of the original model.

A principal component analysis (PCA) revealed a clear distinction between the contents and composition of ethanol extracts prepared from dried (ZZS-G) or fresh (ZZS-S) *P. japonicus* var. *major* samples (Figure 9). Dried samples were clustered on the left side of PC1, and the fresh samples on the right, indicating good separation along the first principal component (PC1). PC1 and PC2 accounted for 69.3% and 19.2% of the total variance, respectively, suggesting a well-structured and reliable model. Partial least squares discriminant analysis (PLS-DA) presented a distinct separation between ZZS-G and ZZS-S samples with tight clustering within each group, reflecting good reproducibility (Figure 10A). The prediction results of the PLS-DA were very close to those of the PCA, which also indicated the strength of the model. The model yielded a *Q*^2^ of 0.997, indicating very good predictability (Figure 10B). Moreover, *Q*^2^ and *R*^2^*Y* both had *p*-values of <0.005, attesting to the statistical significance and reliability of the model.

**Figure 11:**
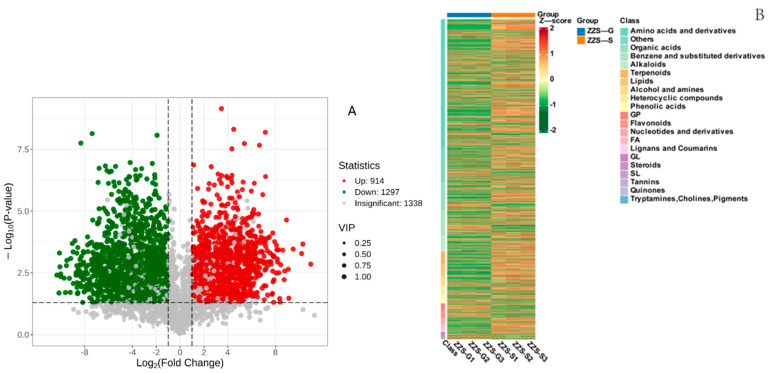
Volcano plot and cluster analysis heatmap of metabolites from *Panax japonicus* var. *major* extracts. (**A**) Volcano plot, with each point representing a metabolite: red indicates upregulated, green downregulated, and gray no significant change in abundance, for a comparison of extract content between ZZS-S and ZZS-G extracts. (**B**) Heatmap representation of metabolite abundance, following a clustering analysis.

A volcano plot analysis of the differentially abundant metabolites in the fresh and dried sample groups revealed that 914 metabolites were significantly upregulated, while another 1297 were significantly downregulated (Figure 11A). Based on a clustering analysis, amino acids and derivatives, organic acids, and alkaloids were the key differentially abundant metabolites between the two groups of *P. japonicus* var. *major* samples (Figure 11B).

**Figure 12:**
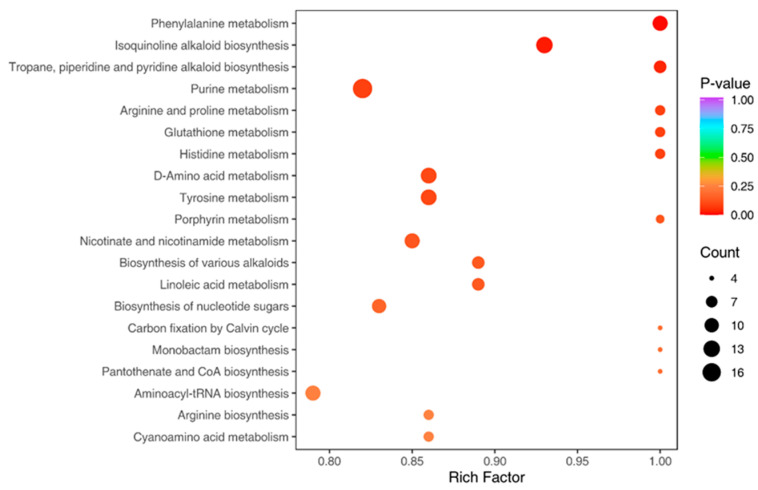
KEGG enrichment analysis of differentially abundant metabolites between ZZS-S and ZZS-G extracts. The Rich factor is the ratio of the number of differentially abundant metabolites in a pathway to the number of annotated metabolites in that pathway. An enrichment greater than one is indicated by a Rich factor greater than one.

We performed a KEGG pathway enrichment analysis based on these differentially abundant metabolites. The most enriched pathways included ‘phenylalanine metabolism’, ‘isoquinoline alkaloid biosynthesis’, ‘tropane, piperidine and pyridine alkaloid biosynthesis’, and ‘arginine biosynthesis’ (Figure 12). These pathways have previously been suggested to be related to gastric cancer [31,32,33,34], suggesting that the observed differences in anti-GC activity between the extracts prepared from fresh or dried *P. japonicus* var. *major* samples may reflect differences in the biosynthesis of these metabolites depending on whether fresh or dried samples were used.

### 2.8. Cell Viability Assays

**Figure 13:**
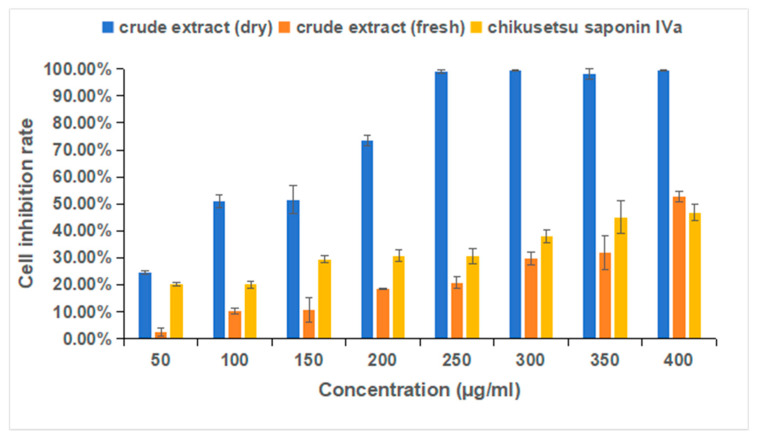
MTS cell (HGC-27) inhibition rate assay. For the crude extracts, each sample was treated with a 75% ethanol extract of *Panax japonicus* var. *major*.

We performed a cell viability assay with HGC-27 gastric cancer cells treated with a crude extract from dry or fresh *P. japonicus* var. *major* samples, or with chikusetsu saponin IVa. Each of these treatments inhibited the proliferation of HGC-27 cells (Figure 13). The inhibitory effects of the two extracts and chikusetsu saponin IVa increased in a dose-dependent manner. The IC_50_ value for the 75% ethanol extract from dried or fresh *P. japonicus* var. *major* samples were 104.6 μg/mL and 440.8 μg/mL, respectively, while that for chikusetsu saponin IVa was 564.7 μg/mL (with biological replicates, *p* < 0.05). These results confirm that both extracts and chikusetsu saponin IVa possess notable anticancer activity against HGC-27 cells. Moreover, the activity of the extract from dry samples was significantly higher than the activity of the extract from fresh samples.

### 2.9. Immunoblot Assays

**Figure 14:**
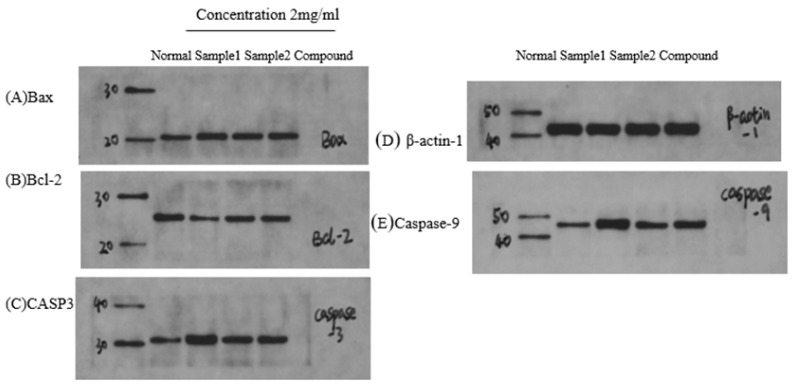

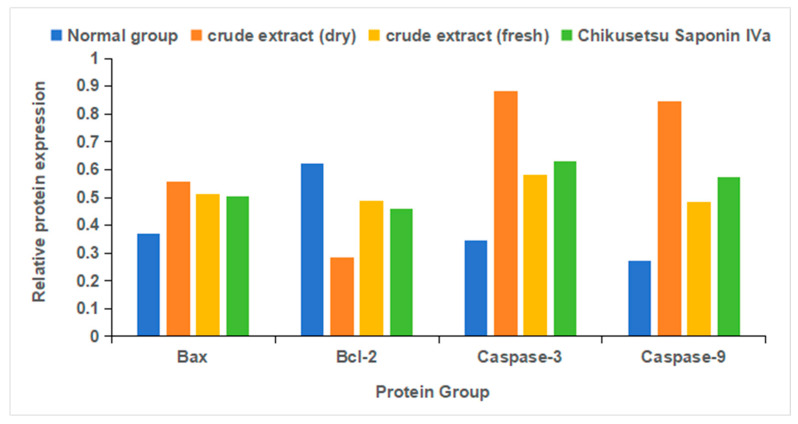
Immunoblot analysis of candidate target proteins upon treatment with *Panax japonicus* var. *major* extracts. The treatment groups were administered at a concentration of 2 mg/mL. Sample 1, extract from dry *P. japonicus* var. *major*; Sample 2, extract from fresh *P. japonicus* var. *major*. (**A**) Bax, (**B**) Bcl-2, (**C**) CASP3, (**D**) β-actin-1, (**E**) caspase 9. The lower plot shows the relative abundance of each protein, normalized to that of β-actin-1.

**Figure 15:**
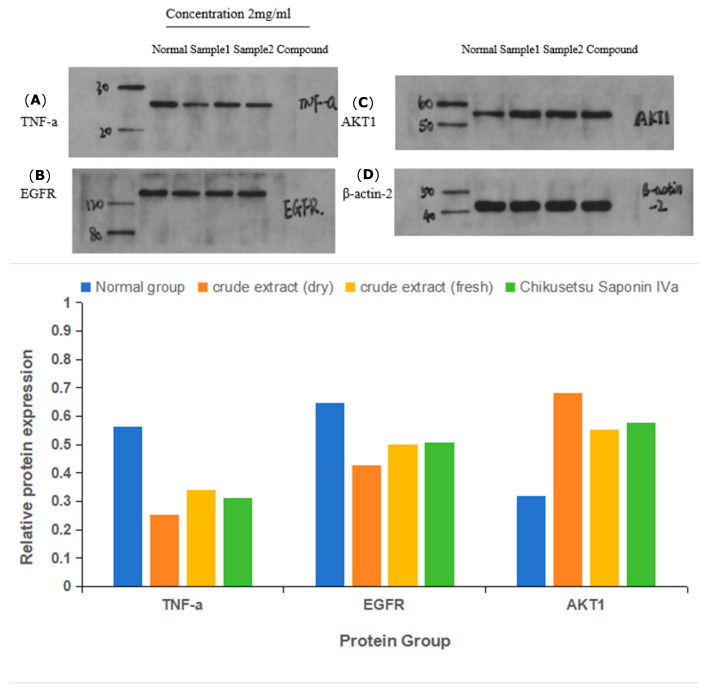
Immunoblot analysis of candidate target proteins upon treatment with *Panax japonicus* var. *major* extracts. The treatment groups were administered at a concentration of 2 mg/mL. Sample 1, extract from dry *P. japonicus* var. *major*; Sample 2, extract from fresh *P. japonicus* var. *major*. (**A**) TNF-a, (**B**) EGFR, (**C**) AKT1, (**D**) β-actin-2. The lower plot shows the relative abundance of each protein, normalized to that of β-actin-2.

Treatment with the 75% ethanol extracts of *P. japonicus* var. *major* samples or chikusetsu saponin IVa significantly induced the accumulation of CASP3 and caspase 9, thereby inducing apoptosis in HGC-27 cells (Figure 14 and Figure 15). Additionally, Bax abundance markedly rose, while that of Bcl-2 declined, further enhancing apoptosis and inhibiting cell proliferation. The abundance of TNF-α was notably downregulated, whereas that of AKT1 and EGFR was elevated following treatment with the compounds.

## 3. Discussion

*P. japonicus* var. *major*, as a traditional medicinal plant, shares similar physical characteristics and medicinal effects with ginseng, possessing a rich medical material foundation and significant medicinal value. Its extracts primarily function to nourish the lungs and Yin, improve blood circulation, relieve pain, and stop bleeding [35], and have gained widespread application in the folk medicine of various ethnic groups. The Naxi people used *P. japonicus* var. *major* as a daily health supplement, commonly made by soaking its rhizome in alcohol or cooking its rhizome in soups to enhance immunity and protect the gastric mucosa [36]. Additionally, this plant can also be used to treat stomach pain, pharyngitis, and swollen sores. The rhizome of *P. japonicus* var. *major* was used by Bai people to treat modern common diseases such as gastric pain and lymphoma, and its use as an antitumor treatment has been recorded in ethnic classic medical books. Pharmacological research showed that certain saponin active compounds contained in *P. japonicus* var. *major* possessed anticancer and liver-protective effects [21], while its small amounts of flavonoids and triterpene saponin compounds exhibited simultaneous anti-inflammatory and antioxidant properties [23]. Overall, while *P. japonicus* var. *major* has demonstrated antitumor efficacy; the underlying mechanisms are complex, involving multiple components and targets.

Network pharmacology is an emerging research methodology that integrates systems biology, bioinformatics, and computer science to construct drug–target–disease networks and reveal the systemic mechanisms of antitumor drugs. This approach can analyze the effective components and targets of traditional Chinese medicine formulations, elucidate the molecular mechanisms of their synergistic antitumor effects, while identifying key tumor nodes and pathways to provide potential targets for new drug development and accelerate drug development [37,38,39].

In this study, using network pharmacology, we predicted the activity of compounds extracted from *P. japonicus* var. *major* against gastric cancer (GC) and explored the potential molecular mechanisms of their anti-GC properties. First, we assembled a list of chemical compounds present in *P. japonicus* var. *major* extracts, yielding 44 active compounds from the ETCM database, with 355 predicted target proteins based on the Swiss Target Prediction database. We separately curated a list of 2103 GC targets from the Gene Cards and OMIM databases. Second, we defined 125 common targets shared between targets of *P. japonicus* var. *major* active compounds and GC targets, which we used to construct a protein–protein interaction (PPI) network. The main core targets obtained from the PPI network, including AKT1, TNF, EGFR, and CASP3, are directly related to apoptosis and are known to cause inflammation in GC cells. Third, we determined the top 10 GO terms and 20 KEGG pathways enriched among the 29 core targets. The core targets were mainly predicted to localize to the cytoplasm and nucleus, where they are expected to bind to other proteins and enzymes [40,41], and have functions related to protein phosphorylation. We determined that *P. japonicus* var. *major* extracts act against GC through the TNF and T-cell receptor signaling pathways by the binding of its characteristic saponins to the core targets TNF and CASP3 in those two signaling pathways, which are highly important in cancer and inflammation [42,43,44]. We found that three compounds interacted with CASP3; one compound interacted with TNF; two compounds interacted with AKT1; and five compounds interacted with EGFR. EGFR is a transmembrane receptor tyrosine kinase with key roles in various aspects of cell biology, including cell proliferation, differentiation, migration, and survival. EGFR has a close relationship with the occurrence, development, and treatment of cancer [45,46]. AKT1 is a core member of the PI3K/AKT/mTOR signaling pathway and exhibits overactivation or mutations (especially the E17K mutation) in various cancers, promoting tumor development through multiple mechanisms, including inhibition of apoptosis, enhancement of cell proliferation, regulation of cellular metabolism, and angiogenesis. The abnormal activation of AKT1 is not only closely associated with tumor progression but also involved in tumor resistance mechanisms to chemotherapy, radiotherapy, and targeted therapies [47].

Based on the results of our network pharmacology analysis, we explored the potential mechanisms of the active compounds in *P. japonicus* var. *major* extracts in the treatment of GC, using molecular docking and other predictions. We chose two compounds (chikusetsu saponin IVa and calenduloside E) with high values as ligands for molecular docking analysis. Chikusetsu saponin IVa is typically used as a quality standard of traditional Chinese medicine [48]. Both compounds were previously reported to show excellent activity against hepatocellular carcinoma [49]. From a compound–target network, we selected the four targets AKT1, TNF, EGFR, and CASP3 for molecular docking analysis. Six of the eight compound–target pairs (Chikusetsu saponin IVa–AKT1, Chikusetsu saponin IVa–CASP3, Chikusetsu saponin IVa–EGFR, calenduloside E–AKT1, calenduloside E–CASP3, and calenduloside E–EGFR) had strong binding characteristics, with the other two pairs (Chikusetsu saponin IVa–TNF, calenduloside E–TNF) showing weaker but still high binding properties. These molecular docking results align with the findings from our network pharmacological analysis.

To evaluate the results of molecular docking predictions, we performed molecular dynamics simulations and free binding energy calculations for the chikusetsu saponin IVa–CASP3 complex, which indicated that the complex was structurally stable, as indicated by the calculated RMSD values. RMSF and SASA calculations for the complex after secondary structure stabilization suggested that the CASP3 surface area becomes smaller following saponin binding. Calculations of H-bonds and binding free energy showed that chikusetsu saponin IVa forms many H-bonds with CASP3 and has a strong binding capacity. The CASP3–chikusetsu saponin IVa complex had an energy minimum mainly focused on 99 ns according to the free energy landscape. The complex was relatively compact and structurally stable in this low-energy region.

To investigate the differences in anti-gastric cancer activity between ethanol extracts prepared from fresh or dried *P. japonicus* var. *major* samples, we conducted a non-targeted metabolomics study on the two crude extracts. There were significant changes in the contents of 2301 differentially abundant metabolites between the dried group (ZZS-G) and fresh group (ZZS-S) of *P. japonicus* var. *major* crude extracts, among which 914 metabolites were significantly upregulated and 1297 significantly downregulated. Among the top 20 metabolites with the highest fold-changes between the two groups were notoginsenoside J and 2-(3,4-dihydroxy-5-methoxyphenyl)-5,6,7-trihydroxy-3,4-dihydro-2*H*-1-benzopyran-4-one, a flavonoid compound. These potential active compounds with anticancer activity require further experimental verification.

To further validate the effects of the *P. japonicus* var. *major* and active compounds identified through binding energy predictions, we conducted in vitro assays. First, the results of an MTS cell viability assay demonstrated that the 75% ethanol extracts of *P. japonicus* var. *major* samples and chikusetsu saponin IVa all effectively suppressed the growth of HGC-27 gastric cancer cells. Moreover, the anti-gastric cancer cell activity of crude extracts from dried *P. japonicus* samples was significantly higher than that from fresh samples. Based on untargeted metabolomics analysis, the significant difference in anti-gastric cancer activity between extracts from fresh and dried samples is likely attributable to the varying abundance of metabolites and to the involvement of specific metabolic pathways. These pathways include phenylalanine metabolism, isoquinoline alkaloid biosynthesis, tropane, piperidine, and pyridine alkaloid biosynthesis, as well as arginine biosynthesis. Changes in the levels of metabolites within these pathways may be closely associated with the observed differences in bioactivity and could have potential implications for the pathophysiology of gastric cancer. Second, immunoblot analysis revealed that these compounds promote apoptosis by increasing the abundance of CASP3 and caspase 9. Furthermore, they modulate the apoptotic regulatory pathway by upregulating Bax and downregulating Bcl-2, thereby enhancing pro-apoptotic activity and inhibiting tumor cell survival. The observed drop in TNF-α abundance suggests that these compounds may also alleviate inflammation, potentially diminishing the risk of inflammation-induced gastric cancer. Collectively, these findings suggest that the 75% ethanol extract of *P. japonicus* var. *major* and chikusetsu saponin IVa hold promise as therapeutic agents for gastric cancer through their dual roles in promoting apoptosis and modulating inflammatory responses. Finally, these findings suggest that *P. japonicus* var. *major* regulates common targets and pathways through these core compounds, thereby exerting therapeutic effects on gastric cancer. In summary, the immunoblot experiments further validated the predictions made through network and molecular docking, providing molecular evidence that *P. japonicus* var. *major* has potential anticancer effects.

In this study, we systematically explored the mechanisms of *P. japonicus* var. *major* for the treatment of GC. Our predictions of the interaction between the *P. japonicus* var. *major* compounds and their GC targets should help provide a theoretical basis for the further development of traditional Chinese medicines. Importantly, clinical trials are needed to further explore the medicinal value of *P. japonicus* var. *major* in cancer. Through network pharmacology, we identified the major active compounds and potential mechanisms of *P. japonicus* var. *major* in anti-gastric cancer activity and experimentally verified its anti-gastric cancer biological activity. This study suggests that the significant difference in anti-gastric cancer cell activity between crude extracts prepared from fresh or dried *P. japonicus* var. *major* samples is related to a series of differentially abundant metabolites between the two sets of extracts and the specific pathways they target. However, whether these differentially abundant metabolites actually possess anti-gastric cancer biological activity and their specific mechanisms of action still require experimental testing. We will conduct research on traditional medicinal plants such as *P. japonicus* var. *major* using different processing methods, while integrating studies on ethnic and folk traditional formulations through multi-omics integrated analysis. We will analyze the complex components of Chinese medicines and their corresponding synergistic mechanisms, thereby providing a theoretical foundation for the development of new drugs.

## 4. Materials and Methods

### 4.1. Medicinal Materials and Chemicals

The medicinal herb *P. japonicus* var. *major* (dried samples and fresh samples) was provided from Xinzhu Village, Ludian Township, Yulong Naxi Autonomous County, Lijiang City, Yunnan Province, China. It was identified by Lixin Yang, a senior engineer at the Kunming Institute of Botany, Chinese Academy of Sciences, Yunnan Province. Chikusetsu saponin IVa reference standard (CAS No.: 51415-02-2) was provided by Shanghai Yuanye Co., Ltd. (Shanghai, China), with a purity greater than 98%. Fresh and dried *P. japonicus* var. *major* samples were extracted with 75% (*v*/*v*) ethanol through soaking, ultrasonic extraction, and rotary evaporation to obtain extracts for experimental and non-targeted metabolomic analysis.

### 4.2. Collection of Main Active Compounds and Corresponding Targets

The known active compounds of *P. japonicus* var. *major* were obtained from The Encyclopedia of Traditional Chinese Medicine database (http://www.tcmip.cn/ETCM/) (accessed on 5 August 2024) [50]. The chemical structures of these active compounds were obtained from the PubChem database (https://pubchem.ncbi.nlm.nih.gov/) (accessed on 5 August 2024). The potential targets of these active compounds were predicted by the Swiss Target Prediction tool (http://www.swisstargetprediction.ch/) (accessed on 6 August 2024) [51].

### 4.3. Collection of Disease-Related Targets

Targets for treating GC were obtained from searches of the databases Gene Cards (https://www.genecards.org/) (accessed on 7 August 2024) [52] and OMIM (https://www.omim.org/) (accessed on 7 August 2024) [53], using the keyword ‘gastric cancer’. The two sets of targets were merged, and redundant targets were removed to define a set of non-redundant targets. A Venn diagram was used to predict the overlap between *P. japonicus* var. *major* target genes and putative targets for GC treatment. The shared 125 targets were considered common targets for GC treatment by compounds from *P. japonicus* var. *major*.

### 4.4. Protein–Protein Interaction (PPI) Network Analysis

Cytoscape 3.10.2 software was used to analyze and visualize the network of potential targets [54]. The common targets defined above were submitted as input to the String database (https://string-db.org) (accessed on 9 August 2024) to generate a gene–protein interaction network [55]. First, the main active ingredients and their corresponding targets were collected into Network files. Second, the network comprising the common targets and active ingredients was exported as Tape files. Third, the network files and Tape files were imported into Cytoscape 3.10.2 to assemble and visualize the compound–target network. The Cytoscape 3.10.2 filtering conditions were set to closeness > 0.046, betweenness > 99.70, and degree > 33.07. The resulting PPI network allowed us to predict the main compounds with potential anti-GC effects and their corresponding core targets.

### 4.5. Gene Ontology Term Enrichment and Kyoto Encyclopedia of Genes and Genomes Pathway Enrichment Analysis

The gene ontology (GO) term and Kyoto Encyclopedia of Genes and Genomes (KEGG) pathway enrichment analysis was carried out on the 29 core targets through the DAVID database (https://david.ncifcrf.gov/) (accessed on 10 August 2024) [56], with the setting ‘*Homo sapiens*’. The corresponding Biological Process (BP), Cellular Component (CC), Molecular Function (MF) terms, and KEGG pathways were analyzed as indicated above and visualized with the ggplot2, openxls, and tidyverse packages in R. The top 20 KEGG pathways were used to construct a component–target–pathway diagram through Cytoscape 3.10.2 combined with the main active ingredients and the core targets of GC.

### 4.6. Survival Analysis

Analysis of the potential effect of inhibiting individual targets on the survival of GC patients was carried out using the online tool Kaplan–Meier Plotter (https://kmplot.com/analysis/) (accessed on 20 August 2024) [57]. Disease type was selected as ‘gastric cancer’, and the species was set to ‘human’. A *p*-value < 0.05 was set as the threshold for statistical significance. Survival analysis was used to predict factors affecting patient survival time. The potential effects of changes in target protein abundance/activity were analyzed according to whether high or low abundance of each protein was beneficial for patient survival.

### 4.7. Molecular Docking Analysis

Molecular Operating Environment 2019 (MOE2019) and PyMol2.6.0 were used for molecular docking and to visualize the top four core targets and the top two compounds. MOE2019 software was used to estimate the energy minimization of compounds. The Protein Data Bank (PDB, https://www.rcsb.org/) (accessed on 1 September 2024) [58] was used to download protein structures in the correct format for this analysis. The 3D structures of the active compounds were downloaded from the PubChem database (https://pubchem.ncbi.nlm.nih.gov/) (accessed on 2 September 2024). The format of these structure files was transformed to mol2 format through the OpenBabel Gui software 3.1.1. The calculation of energy minimization for each compound was performed using MOE2019 software. Finally, molecular docking was conducted with MOE 2019 software and 50 operations. The binding activity was assessed based on the magnitude of binding energy, and the results were visualized in PyMOL2.6.0 and Discovery studio 2019 software.

### 4.8. Molecular Dynamics Simulations and Calculation of Binding Free Energies

Gromacs2022.3 software [59,60] was used for molecular dynamics simulations. For small molecule preprocessing, amberTools22 was used to add the GAFF force field to the small molecules, and Gaussian 16W was used to simulate the hydrogenation of small molecules and calculate their RESP potential. The potential data were added to the topology file of the molecular dynamics system. The simulation conditions were a constant temperature of 300 K and atmospheric pressure (1 bar). Amber99sb–ildn was used as a force field, and water molecules were used as solvent (Tip3p water model), and the total charge of the simulation system was neutralized by adding an appropriate number of sodium (Na^+^) ions. The simulation system adopted the steepest descent method to minimize the energy, and then carried out isothermal isovolumic ensemble (moles, volume, temperature [NVT]) equilibrium and isothermal isobaric ensemble (moles, pressure, temperature [NPT]) equilibrium for 100,000 steps, respectively, with a coupling constant of 0.1 ps and a duration of 100 ps. Finally, a free molecular dynamics simulation was performed. The procedure consisted of 5,000,000 steps, with a step length of 2 fs and a total duration of 100 ns. After the simulation was completed, the built-in tool of the software (Gromacs 2022.3 software) was used to analyze the trajectory, and the root-mean-square variance (RMSD), root-mean-square fluctuation (RMSF), and protein rotation radius of each amino acid trajectory were calculated and combined with the free energy (Molecular Mechanics—Generalized Born—Surface Area [MMGBSA]), free energy topography, and other data.

### 4.9. Untargeted Metabolomics

To compare the differentially abundant metabolites and determine the anti-gastric cancer activity of fresh *P. japonicus* var. *major* and its dried, saponin-enriched counterpart, non-targeted metabolomics analysis was conducted with crude extracts in 75% (*v*/*v*) ethanol (ZZS-G group, crude extract from dried *P. japonicus* var. *major*; ZZS-S, crude extract from fresh *P. japonicus* var. *major*). To each sample, 600 μL of 70% (*v*/*v*) methanol was added along with an internal standard solution. The internal standard was prepared by dissolving 1 mg of reference material in 1 mL of 70% (*v*/*v*) methanol to obtain a stock solution (1000 μg/mL), which was diluted to a working concentration of 250 μg/mL. The solution was centrifuged at 12,000 rpm for 3 min, and the supernatant was filtered through a 0.22-μm microporous membrane and transferred to autosampler vials for UPLC-MS/MS analysis. Chromatographic separation was achieved with a Waters ACQUITY UPLC HSS T3 column (1.8 µm, 2.1 mm × 100 mm). The mobile phase consisted of 0.1% (*v*/*v*) formic acid in ultrapure water (solvent A) and 0.1% (*v*/*v*) formic acid in acetonitrile (solvent B). The column temperature was 40 °C, the flow rate was 0.40 mL/min, and the injection volume was 4 µL.

### 4.10. MTS Cell Inhibition Rate

A single-cell suspension (HGC-27 cells [human gastric cancer cell line]) was prepared in RPMI 1640 culture medium supplemented with 10% (*v*/*v*) fetal bovine serum (FBS). A total of 5000 cells were seeded into each well of a 96-well plate, with a final volume of 100 μL per well. The cells were incubated for 12–24 h to allow for adherence prior to treatment. The 75% ethanol extracts of *Panax japonicus* var. *major* served as test sample 1 (dry group) and sample 2 (fresh group); chikusetsu saponin IVa compound (Purchased from Shanghai Yuanye Company, Shanghai, China) was dissolved in DMSO and initially screened at concentrations of 400 μg/mL, 350 μg/mL, 300 μg/mL, 250 μg/mL, 200 μg/mL, 150 μg/mL, 100 μg/mL, and 50 μg/mL. A final volume of 200 μL, containing the compound, was added to each well. Each treatment was performed in triplicate. Following 48 h of incubation at 37 °C, 20 μL of MTS solution and 100 μL of culture medium were added to each well. Blank control wells were included, and the cells were incubated for an additional 2–4 h to ensure complete reaction. The absorbance was subsequently measured at 492 nm using a microplate reader. Doxorubicin (Dox) and paclitaxel (Taxol) were used as positive controls.

### 4.11. Immunoblotting

HGC-27 cells were seeded at a density of 2 × 10^5^ cells/mL in 6-well plates and incubated for 24 h; the drug concentration was 2 mg/mL. After incubation, the cells were divided into treatment groups and cultured for an additional 24 h. Subsequently, the cells were washed twice with ice-cold phosphate-buffered saline (PBS), and total protein was extracted using RIPA lysis buffer. Following denaturation, 20 μL of the protein samples were loaded into each well of an SDS-PAGE gel for electrophoresis. Proteins were transferred onto a PVDF membrane, which was then blocked with blocking buffer at room temperature for 2 h under gentle agitation. The membrane was incubated with primary antibodies overnight at 4 °C and then for 2 h with a secondary antibody at room temperature. After washing with PBS containing 0.1% (*v*/*v*) Tween-20 (PBST), the membrane was developed using enhanced chemiluminescence (ECL) reagents. The membrane was air-dried, and the resulting images were scanned. Protein band intensities were quantified based on grayscale values using Image-Pro Plus (IPP) software 7.0.

## 5. Conclusions

In this study, we used network pharmacology to identify the main active compounds of *P. japonicus* var. *major* extracts and their potential targets in relation to the treatment of gastric cancer (GC). A protein–protein interaction network allowed us to explore the interaction between the main compounds of *P. japonicus* var. *major* extracts and their putative targets related to GC, as well as their potential mechanisms of action against GC. Predictions from network pharmacology suggest that chikusetsu saponin IVa and calenduloside E have good anti-GC activity, which was supported by survival analysis assays and molecular docking simulations, combined with molecular dynamics simulations and calculations of binding free energy. We assessed the structural stability, energy nadir, and change in binding energy of small drug molecules bound to four target proteins. We verified the anti-gastric cancer activity and mechanism of crude ethanol extracts and chikusetsu saponin IVa through MTS cell inhibition assay and immunoblots. Through the application of untargeted metabolomics, we analyzed the differential abundance of metabolic products of *P. japonicus* var. *major* to explore the factors contributing to the differences in anti-GC activity between extracts prepared from fresh and dried samples. We carried out a comprehensive analysis of the binding possibilities and accuracy of the molecules revealed by the simulations, which provided insight into the potential anti-GC effects and mechanisms of action for chikusetsu saponin IVa of *P. japonicus* var. *major*.

## Figures and Tables

**Table 1:** The top 10 active compounds based on diminishing degree values from *P. japonicus* var. *major*.

Compound Name	Compound Structure	Degree	Betweenness	Closeness
Panaxjapyne C	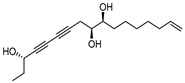	45.0	8980.48	0.23108666
Panaxjapyne B	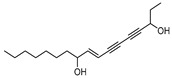	45.0	8888.03	0.22919509
Cholesterol	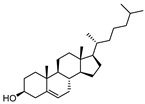	22.0	2596.21	0.20512821
β-Sitosterol	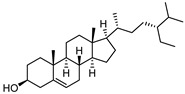	19.0	1565.01	0.20363636
Stigmasterol	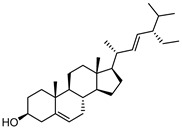	17.0	1001.37	0.20071685
Notoginsenoside R2	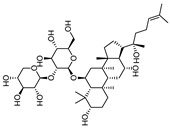	13.0	759.52	0.19421965
3,4,5-Trimethoxybenzoic Acid	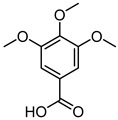	12.0	3169.00	0.19156215
Chikusetsusaponin IVa	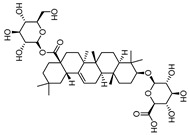	12.0	1601.09	0.20664206
Stipuleanoside R2	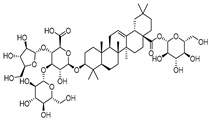	11.0	739.68	0.20023838
Calenduloside E	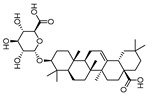	11.0	782.38	0.20715167

Note: Degree, betweenness, and closeness values are based on the network shown in Figure 2A.

**Table 2:** The top 29 gastric cancer target proteins ranked by their degree value in the PPI network.

Protein Name	Degree	Protein Name	Degree
AKT1	96	MAPK14	61
TNF	93	GSK3B	59
EGFR	87	MAPK1	59
CASP3	84	KDR	58
STAT3	84	EP300	58
JUN	83	HRAS	58
ESR1	78	FGF2	56
HSP90AA1	74	PARP1	54
ERBB2	73	IL2	53
MMP9	73	ICAM1	48
MAPK3	69	CDK4	47
MTOR	69	ABL1	46
PTGS2	67	AR	44
PPARG	67	ACE	34
PIK3CA	62		

Note: Degree values are based on the network shown in Figure 2B.

**Table 3:** Binding free energy of the indicated complexes.

	CASP3–Chikusetsu Saponin IVa
Van der Waals force	−70.74 ± 1.28
*E*_EL_	−54.74 ± 1.96
*E*_GB_	78.59 ± 4.05
*E*_SURF_	−9.88 ± 0.09
Δ*G*_GAS_	−125.48 ± 2.34
Δ*G*_SOLV_	68.70 ± 4.05
ΔTotal	−56.77 ± 4.68

## Data Availability

Data are contained within the article and Supplementary Materials.

## Supplementary Materials

The following supporting information can be downloaded at: https://www.mdpi.com/article/10.3390/ph18060823/s1, Figure S1: Main active compounds; Table S1: HGC-27 cell information.

## Abbreviations

GC	Gastric cancer
ETCM	Encyclopedia of Traditional Chinese Medicine
KEGG	Kyoto Encyclopedia of Genes and Genomes
GO	Gene Ontology
MD	Molecular Dynamics
RMSD	Root-Mean-Square Deviation
RMSF	Root-Mean-Square Fluctuation
MMPBS	Molecular Mechanics Poisson-Boltzmann Surface Area
FBS	Fetal Bovine Serum
ECL	Enhanced Chemiluminescence
IPP	Image-Pro Plus
ZZS-G	Dried *P. japonicus* var. *major* Group
ZZS-S	Fresh *P. japonicus* var. *major* Group
