# Supplementary material for: Molecular Mechanisms of Panax japonicus var. major Against Gastric Cancer: Metabolite Analysis, Signaling Pathways, and Protein Targets
Source: Pharmaceuticals (Basel). 2025 May 30;18(6):823. doi: 10.3390/ph18060823 (PMC12196413; doi:10.3390/ph18060823)
Supplement: Supplementary file 1 [file pharmaceuticals-18-00823-s001.zip › Figure S1(Main active compounds).pdf]

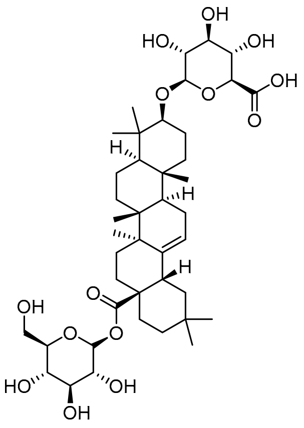

Chikusetsu saponin IVa

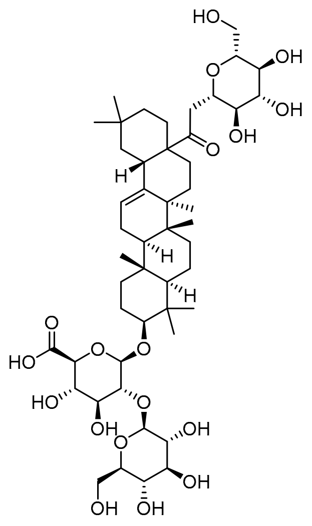

Ginsenoside Ro

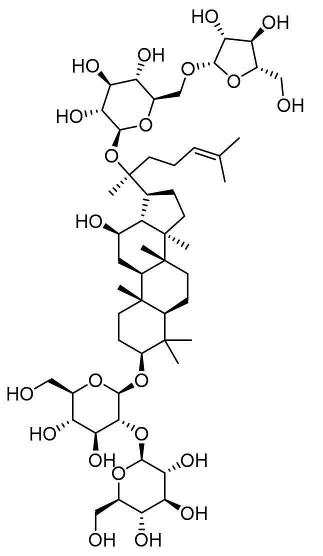

Ginsenoside Rc

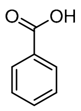

Benzoic Acid

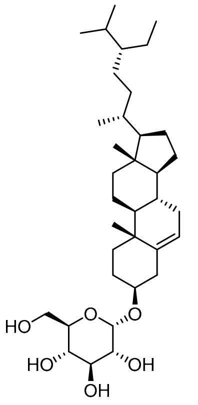

Daucosterol

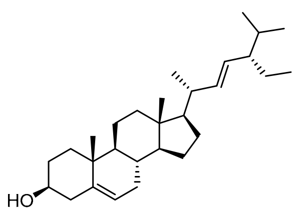

Stigmasterol

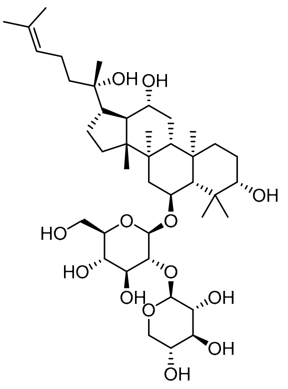

Notoginsenoside-R_2

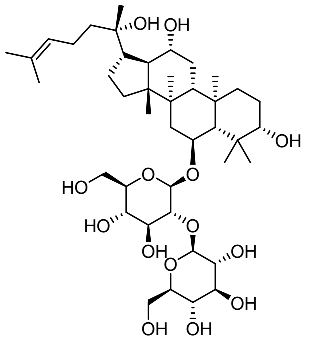

Ginsenoside Rf

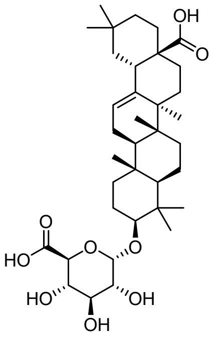

Calenduloside E

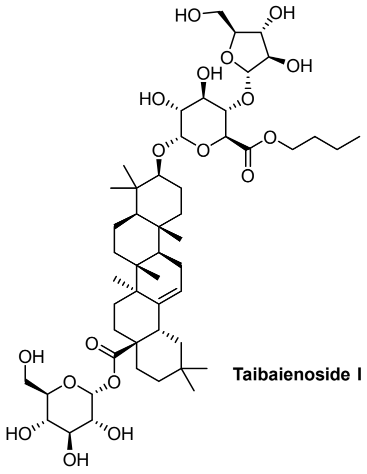

Taibaienoside I

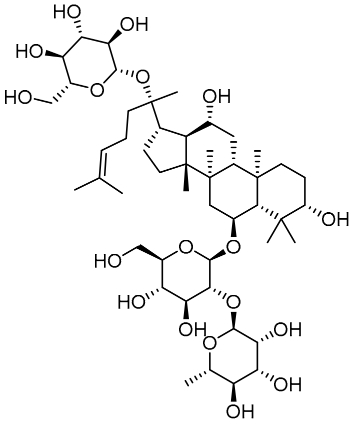

Ginsenoside Re

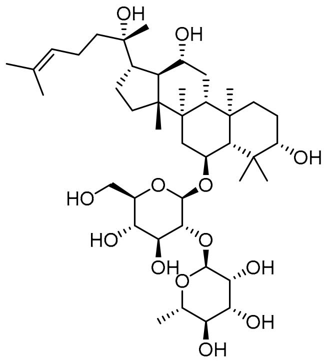

Ginsenoside Rg_2

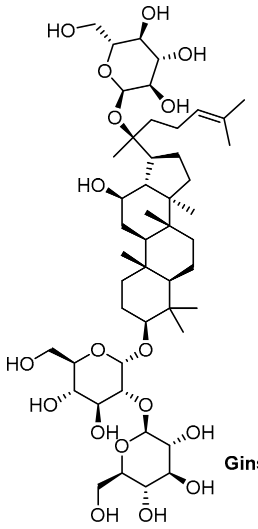

Ginsenoside Rd

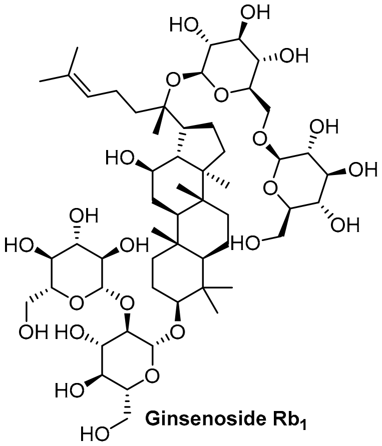

Ginsenoside Rb_1

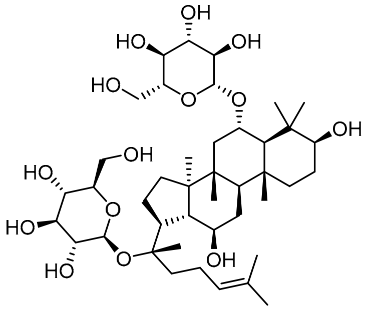

Ginsenoside Rg_1

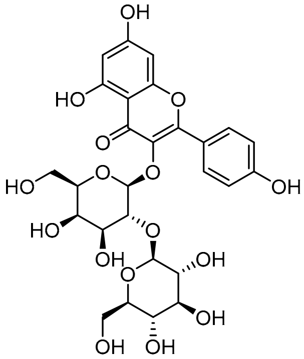

Panasenoside

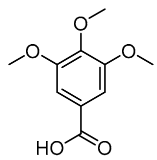

3,4,5-Trimethoxybenzoic Acid

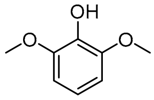

2,6-Dimethoxyphenol

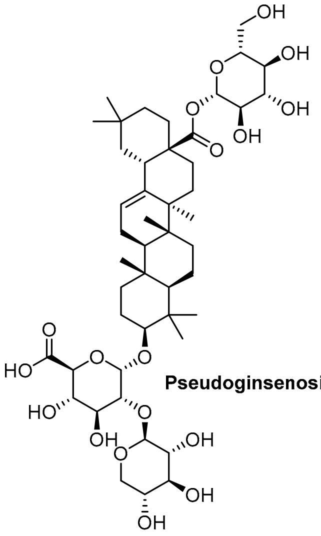

Pseudoginsenoside Rt_1

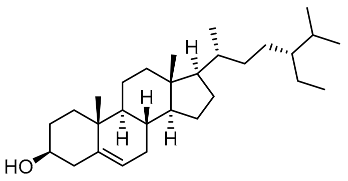

β-Sitosterol

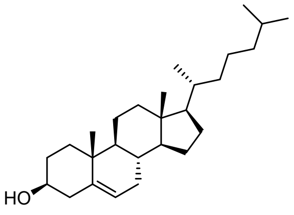

Cholesterol

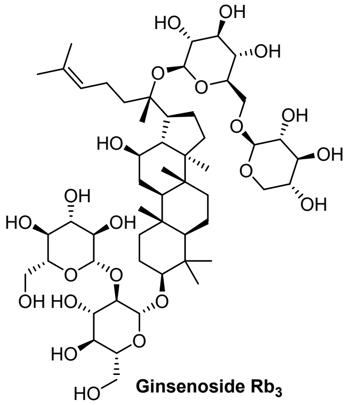

Ginsenoside Rb_3

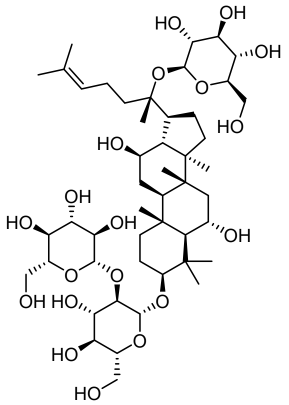

Vinaginsenoside R_4

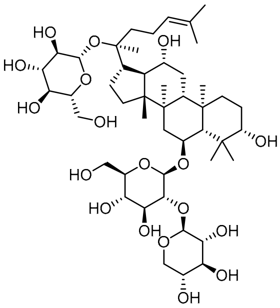

Notoginsenoside R_1

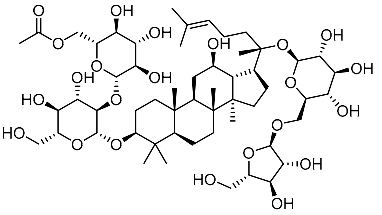

Ginsenoside Rs_2

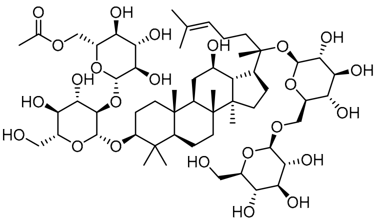

Quinquenoside R_1

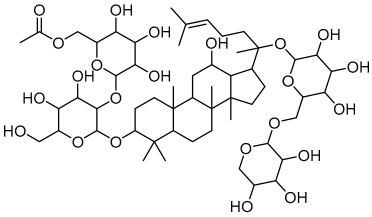

Ginsenoside Rs_1

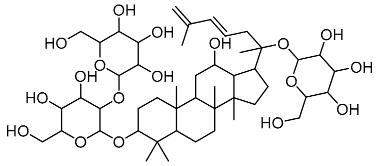

Quinquenoside L_1

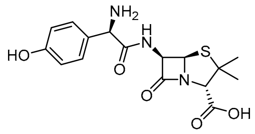

Notoginsenoside-Fe

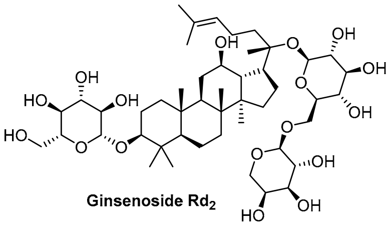

Ginsenoside Rd_2

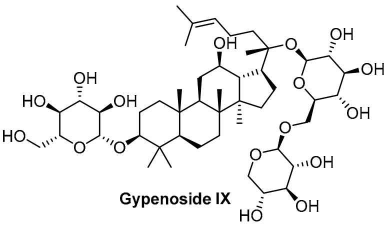

Gypenoside IX

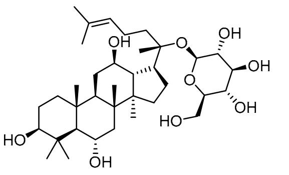

Ginsenoside F_1

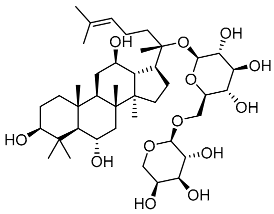

Ginsenoside F_3

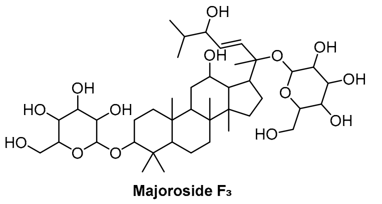

Majoroside F_3

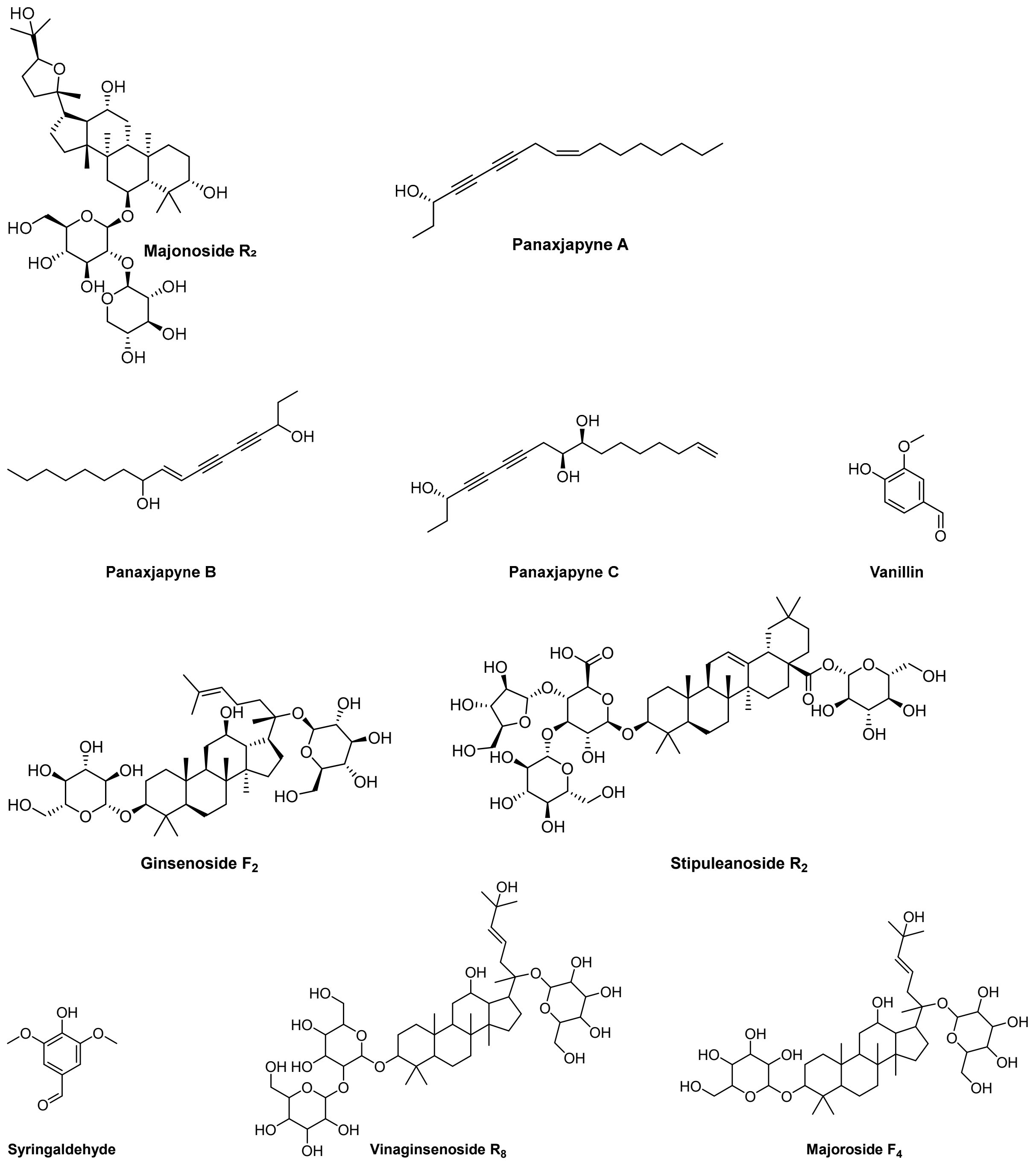

Figure S1. Main active compounds.
