# Supplementary material for: Molecular Mechanisms of Panax japonicus var. major Against Gastric Cancer: Metabolite Analysis, Signaling Pathways, and Protein Targets
Source: Pharmaceuticals (Basel). 2025 May 30;18(6):823. doi: 10.3390/ph18060823 (PMC12196413; doi:10.3390/ph18060823)
Supplement: Supplementary file 1 [file pharmaceuticals-18-00823-s001.zip › Table S1 (HGC-27 cell information).pdf]

Table S1: HGC-27 cell information.
HGC-27 cells were purchased from Qisai Biotechnology (Wuhan) Co., Ltd.

Genotyping results of STR loci and Amelogenin locus of cells

Loc	STR information of submitted cells for testing			STR information of cell bank cells		
	Name of submitted cells for testing：HGC-27			Name of cell bank cells：HGC-27		
	Allele1	Allele2	Allele3	Allele1	Allele2	Allele3
D5S818	12	12		12	12	
D13S317	10	11		10	11	
D7S820	11	12	13	11	12	13
D16S539	10	11		10	11	
VWA	14	14		14	14	
TH01	9	9		9	9	
AMEL	X	X		X	X	
TPOX	8	8		8	8	
CSF1PO	12	12		12	12	
D12S391	20	21				
FGA	22	22				
D2S1338	22	24				
D21S11	30	33	34			
D18S51	16	17				
D8S1179	11	11				
D3S1358	17	17				
D6S1043	18	18				
PENTAE	17	18				
D19S433	14	14				
PENTAD	9	13				
D1S1656	14	16				
